# Immunosenescence accelerates atherosclerosis development in AAV-PCSK9 mouse model

**DOI:** 10.1007/s11357-025-01768-6

**Published:** 2025-07-03

**Authors:** Jill de Mol, Virginia Smit, Mireia N. A. Bernabé Kleijn, Peter J. van Santbrink, Ilze Bot, Amanda C. Foks

**Affiliations:** Division of BioTherapeutics, Leiden Academic Centre for Drug Research, Leiden University, Einsteinweg 55, 2333 CC Leiden, The Netherlands

**Keywords:** Cardiovascular disease, Atherosclerosis, Aging, Immunology, Immunosenescence

## Abstract

Aging is a dominant risk factor for atherosclerotic cardiovascular disease and is associated with compositional and functional changes in our immune system, called immunosenescence. To gain insights into the impact of immunosenescence on atherosclerosis, we investigated plaque development and the immune landscape in an AAV-PCSK9 mouse model. Young (3 months) and aged (18 months) male C57Bl/6 mice received a single i.v. injection of a recombinant adeno-associated virus encoding murine PCSK9 (rAAV8-D377Y-mPCSK9) and were fed a Western-type diet (WTD) for 10 weeks to induce atherosclerosis. At sacrifice, cholesterol and serum PCSK9 levels showed no difference. Atherosclerosis development in the aortic root was significantly enhanced with 59% in aged compared to young mice. Additionally, plaques in aged mice showed a more advanced phenotype, with an increased collagen/macrophage ratio. While the T cell percentage declined in the periphery and lymphoid organs upon aging, leukocytes, including T- and B cells, increased within the aged aortic arch. Moreover, we observed a shift towards effector (memory) subsets, including Th1 cells and Tregs, with elevated cytokine production and a senescent phenotype within the T cell compartment of aged mice. We also observed increased percentages of splenic age-associated B cells, regulatory B cells and plasma cells in aged mice, which coincided with increased levels of circulating antibodies. In conclusion, aged WTD-fed AAV-PCSK9 mice displayed pro-atherogenic immune alterations, including elevated plaque inflammation, increased cellular senescence and impaired humoral immune responses, contributing to accelerated atherosclerosis development compared to young mice. Targeting or reversing age-associated immunity could represent a promising strategy to combat atherosclerotic cardiovascular disease.

**Graphical Abstract:**

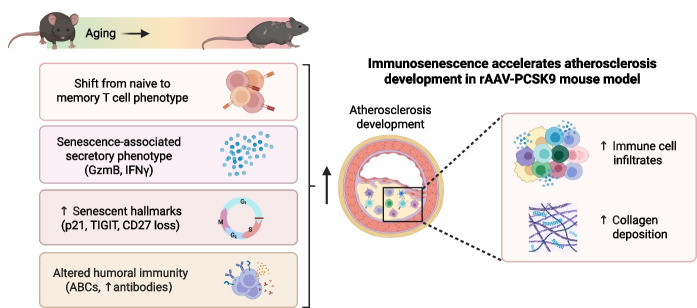

**Supplementary Information:**

The online version contains supplementary material available at 10.1007/s11357-025-01768-6.

## Introduction

The ongoing demographic shift towards an older population results in increased morbidity and mortality from age-associated diseases including atherosclerotic cardiovascular disease (CVD) [1, 2]. Atherosclerosis is a chronic inflammatory disease which is characterized by the formation of lipid-rich lesions in medium- to large-sized arteries [3]. In response to lipid accumulation, monocytes and lymphocytes are recruited to the endothelial cell layer. Upon migration into the vessel wall, monocytes differentiate into macrophages that can phagocytose lipids and form foam cells. T and B lymphocytes mediate foam cell formation and atherogenesis, mainly via the secretion of cytokines and antibodies [4]. Pro-atherogenic T helper 1 (Th1) cells secrete interferon (IFN)-γ and tumor necrosis factor (TNF)-α, which promote the expression of adhesion molecules on endothelial cells, the activation of macrophages and hypertriglyceridemia, thereby aggravating atherosclerosis [5–7]. In contrast, regulatory T cells (Tregs), exert atheroprotective effects by producing anti-inflammatory cytokines, including IL-10 and TGF-β [8], which suppress effector T cell responses and limit plaque progression [9, 10]. The roles of Th2, Th9 and Th17 cells are less well understood. Th2 cells may attenuate Th1-driven inflammation through IL-4, IL-5 and IL-13 [11–13], but excessive Th2 activity may promote fibrosis [14]. Th9 and Th17 cells secrete IL-9 and IL-17, respectively, which have been associated with pro-inflammatory responses and immune cell recruitment but, in the case of Th17, also plaque stabilization [15–17]. B cells can halt foam cell formation by the production of IgM antibodies [18], but can also promote atherosclerosis development by the stimulation of monocyte infiltration, IgG production and Th1 skewing [19]. Acute cardiovascular events such as myocardial infarction or stroke occur upon rupture or erosion of advanced plaques.

Aging is associated with a reduced lymphoid output of the bone marrow and a functional decline in the immune system, termed immunosenescence, which may contribute to the elevated risk for atherosclerosis. These immunosenescent changes include decreased phagocytic ability of macrophages, altered (auto-)antibody production by B cells, and accumulation of senescent cells that can secrete a variety of pro-inflammatory cytokines, chemokines, and proteases [20, 21]. These changes can contribute to an age-associated low-grade chronic inflammation, known as inflammaging, which might contribute to the accelerated chronic inflammation in CVD patients [22, 23]. The senescent phenotype of T cells, which is mainly characterized by the lack of the co-stimulatory molecule CD28 [24], is increased in coronary artery disease patients [25], and the presence of these CD28^null^ CD4^+^ and/or CD8^+^ T cells is associated with an elevated CVD risk [26–29]. In addition, the frequency of T cells expressing CD57, which is a marker of terminal differentiation [30], is elevated in CVD patients [25, 31]. Although studies investigating immunosenescence and inflammaging in atherosclerosis are scarce, we have recently shown that aging of *Ldlr*^*−/−*^ mice resulted in the gradual development of highly advanced, calcified and fibrotic plaques with large immune infiltrates [32, 33]. More specifically, we identified age-associated T- and B cells with pro-inflammatory signatures in atherosclerotic aortas of aged *Ldlr*^*−/−*^ mice and in the circulation and plaques of CVD patients.

Unlike aging of *Ldlr*^*−/−*^ and *ApoE*^*−/−*^ mice, in which atherosclerosis and aging progress in parallel, the AAV-PCSK9 model allows us to induce atherosclerosis in young versus aged C57Bl/6 mice upon an acute elevation of plasma lipids and to investigate the impact of immunosenescence on atherosclerosis development. In this study, we therefore employed the AAV-PCSK9 mouse model of atherosclerosis to investigate the impact of aging on plaque formation, composition and the immune landscape. We found aggravated atherosclerosis development in aged AAV-PCSK9 mice, with elevated plaque inflammation, increased cellular senescence, and impaired humoral immunity compared to young AAV-PCSK9 mice, contributing to a pro-atherogenic environment.

## Methods

### Animals

All animal experiments were approved by the Leiden University Animal Ethics Committee and were performed according to the guidelines of the European Parliament Directive 2010/63/EU of the European Parliament. Male C57Bl/6 J mice (3 months old) were bred in-house and aged male C57Bl/6 J mice (18 months) were purchased at the age of 14 months from Charles River (Sulzfled, Germany) and further aged in-house. All mice were kept under standard laboratory conditions. Mice were fed a Western-type diet (WTD) containing 0.25% cholesterol and 15% cocoa butter (Special Diet Services, UK). Diet and water were provided ad libitum. During the experiment, the health status of the mice was assessed weekly by body condition scoring. This non-invasive method evaluates body condition on a scale from 1 (emaciation) to 5 (obese), based on both visual inspection and palpation, and serves as a key tool for determining humane endpoints when body weight is not a sufficient indicator. At the end of the experiment, mice were euthanized by a subcutaneous injection of a ketamine (100 mg/kg), atropine (0.15 mg/kg), and xylazine (10 mg/kg). Mice were bled by retro-orbital bleeding and tissues were harvested after in situ perfusion with phosphate-buffered saline (PBS).

### PCSK9-induced atherosclerosis

To induce atherosclerosis in young and aged WT mice, 3 months-old (*n* = 15) and 18 months-old male mice (*n* = 12) were administered a single i.v. injection of rAAV2/8-D377Y-mPCSK9 (10 × 10^11^ genome copies/mouse). This results in the rapid overexpression of proprotein convertase subtilisin/kexin type 9 and a significant decrease in hepatic low-density lipoprotein receptor (ldlr) [34]. For atherosclerosis development, mice were subsequently fed a WTD for 10 weeks before mice were sacrificed and relevant organs were harvested for analysis. We excluded 2 mice from the experiment that did not reach serum cholesterol levels > 250 mg/dL upon AAV-PCSK9 administration. One aged mouse died during the course of the experiment.

### Serum cholesterol, PCSK9 level and immunoglobulin measurements

Blood samples were centrifuged at high-speed (10,000 rpm) and serum was collected and frozen at −80 °C until further use. To determine total cholesterol levels, serum samples underwent enzymatic colorimetric procedures (Roche/Hitachi, Germany) with precipath (Roche/Hitachi) as an internal standard. Serum levels of PCSK9 were measured by ELISA according to manufacturer’s protocol (R&D systems). The level of circulating IgA, IgM, IgG1, IgG2b, IgG2b and IgG3 were determined using a Legendplex (Mouse Immunoglobulin Isotyping 6-plex, Biolegend, The Netherlands) and measured on a Cytoflex S (Beckman Coulter, USA).

### Histology

Hearts were embedded in O.C.T. compound (Sakura) and snap-frozen. To determine lesion size, cryosections (10 µm) of the aortic root were stained with Oil-Red-O and hematoxylin (Sigma-Aldrich, the Netherlands). The average of five sequential sections of the three-valve area of aortic roots, with a distance of 80 µm between the sections, displaying the highest lesion content, were used to compare the lesion size (µm^2^). Three sequential sections, again displaying the highest lesion content, were used for plaque composition analysis. Collagen content in the lesions was quantified using a Picro Sirius Red staining (Abcam, UK). Corresponding sections on separate slides were stained for monocyte/macrophage content with a MOMA-2 antibody (1:1000, AbD Serotec, Luxembourg) followed by a biotinylated goat anti-rat IgG antibody (1:200, Vector, Germany). Secondary antibodies were detected using the Vectastain ABC kit (Vector) and visualized with ImmPACT NovaRED HRP substrate (Vector). Pictures were taken with a Panoramic 250 Flash III slide scanner (3DHISTECH, Hungary). Stained sections were manually analyzed with ImageJ software. Analysis was performed blinded.

### Flow cytometry

To obtain single cell suspensions, paraaortic lymph nodes and spleens were mashed through 70 µm cell strainers (Greiner Bio-One, the Netherlands). Erythrocytes in blood and spleen samples were removed with ACK lysis buffer (0.15 M NH4Cl, 1 mM KHCO3, 0.1 mM Na2EDTA; pH 7.3). Aortic arches were digested with 450 U/mL collagenase I, 250 U/mL collagenase XI, 120 U/mL DNAse, and 120 U/mL hyaluronidase for 30 min at 37 °C, and subsequently strained through 70 µm cell strainers. Secretion of cytokines by splenic T cells was induced with stimulation of PMA (50 ng/ml), ionomycin (500 ng/ml), and brefeldin A (5 µg/ml) for 5 h at 37 °C. For flow cytometry analysis, Fc receptors of single cell suspensions were blocked with an unconjugated antibody against CD16/CD32 (clone 2.4G2, BD Biosciences, USA). Samples were then stained with a fixable viability marker (Thermo Fisher Scientific, USA) to select live cells and with anti-mouse fluorochrome-conjugated antibodies (Supplemental Table 1). Antibody staining of transcription factors and cytokines was performed using transcription factor fixation/permeabilization concentrate and diluent solutions and cytofix/permeabilization solutions, respectively (BD Biosciences). Flow cytometry analysis was performed on a Cytoflex S (Beckman Coulter, USA) or LSR Fortessa (BD Biosciences) and the acquired data were analyzed using FlowJo software 8.0.

### Real-time quantitative PCR

RNA was extracted from isolated splenocytes by using Trizol reagent according to manufacturer’s instructions (Invitrogen, USA) after which cDNA was generated using RevertAid M-MuLV reverse transcriptase according to manufacturer’s protocol (Thermo Scientific). Quantitative gene expression analysis was performed using Power SYBR Green Master Mix on a 7500 Fast Real-Time PCR system (Applied Biosystems, USA). Gene expression of p16, p21 and p53 were normalized to housekeeping genes 36B4, Ppia and actin-beta (Supplemental Table 2).

### Statistical analysis

Data are expressed as mean ± SEM. Outliers were identified and removed using Grubbs outlier tests (a = 0.05). Significance was tested using a Student’s t -test for two normally distributed groups or a Mann–Whitney U test for nonparametric groups. *P*-values of < 0.05 were considered significant. Statistical analysis was performed using GraphPad Prism 9.0.

## Results

### Increased atherosclerosis development with a more advanced phenotype in aged mice

**Fig. 1:**
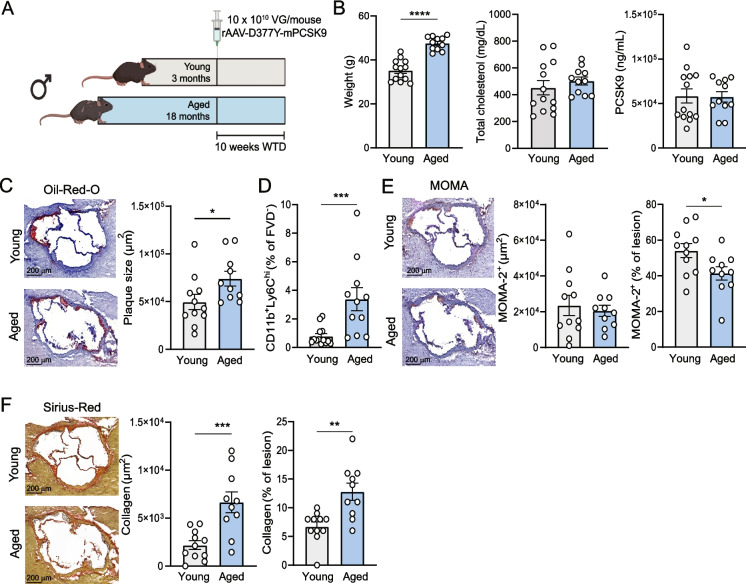
Aged WT mice show increased atherosclerosis development. (**A**) Experimental setup: male C57Bl/6 mice aged 3 months (12 weeks; grey bars) or 18 months (80 weeks; blue bars) were administered a single i.v. injection of rAAV2/8-D377Y-mPCSK9 and subsequently fed a Western diet (WTD) for 10 weeks to induce atherosclerosis. **(B)** Body weight, serum cholesterol levels and circulating PCSK9 levels were measured after 10 weeks. **(C)** Lesion size in the aortic root was determined using an Oil-Red-O and hematoxylin staining. **(D)** Flow cytometry was performed to measure the frequency of circulating Ly6C^hi^ monocytes after 10 weeks. **(E)** Macrophage/monocyte content was assessed with a MOMA-2 antibody and **(F)** collagen content was determined using a Pico Sirius Red staining. Representative pictures are shown, magnitude 5X. Data are from *n* = 11–13 mice per group. Mean ± SEM plotted. *P < 0.05, **P < 0.01, ***P < 0.001, ****P < 0.0001. Created in BioRender. De Mol, J. (2025) https://BioRender.com/rycs4yy

To investigate the impact of immunosenescence on atherosclerosis development, we administered a single injection of a recombinant adeno-associated virus encoding for a gain-of-function for PCSK9 in combination with a Western-type diet for 10 weeks to young (3 months) and aged (18 months) C57Bl/6 mice (Fig. 1A). This resulted in the rapid induction of total cholesterol and circulating PCSK9 levels (Fig. 1B). Although aged mice showed significantly higher body weights, cholesterol and PCSK9 levels were comparable between both age groups. Aged mice displayed 59% increased lesion development compared to their young counterpart (young: 4.64 ± 0.80 × 10^4^ µm^2^ vs. aged: 7.41 ± 0.78 × 10^4^ µm^2^, *p* < 0.05) (Fig. 1C). While we observed elevated levels of circulating Ly6C^high^ monocytes in aged mice (young: 0.79 ± 0.18% vs. aged: 3.38 ± 0.80%, *p* < 0.001) (Fig. 1D), this did not translate in a difference in absolute macrophage area within the lesions (Fig. 1E). Lesions from aged mice did show a 24% decrease in relative macrophage content (young: 54.2 ± 3.91% vs. aged: 41.4 ± 3.77%, *p* < 0.05) (Fig. 1E), attributed to an enrichment in collagen content, both absolute (young: 2.18 ± 0.46 × 10^3^ µm^2^ vs. aged: 6.65 ± 0.11 × 10^3^ µm^2^, *p* < 0.001) and relative (young: 6.73 ± 0.81% vs. aged: 12.80 ± 1.50%, *p* < 0.01), compared to young lesions (Fig. 1F). Altogether, this data shows accelerated atherosclerosis development in aged mice with a more progressed plaque phenotype.

### Elevated numbers of immune cells in atherosclerotic aortic arch of aged mice

**Fig. 2:**
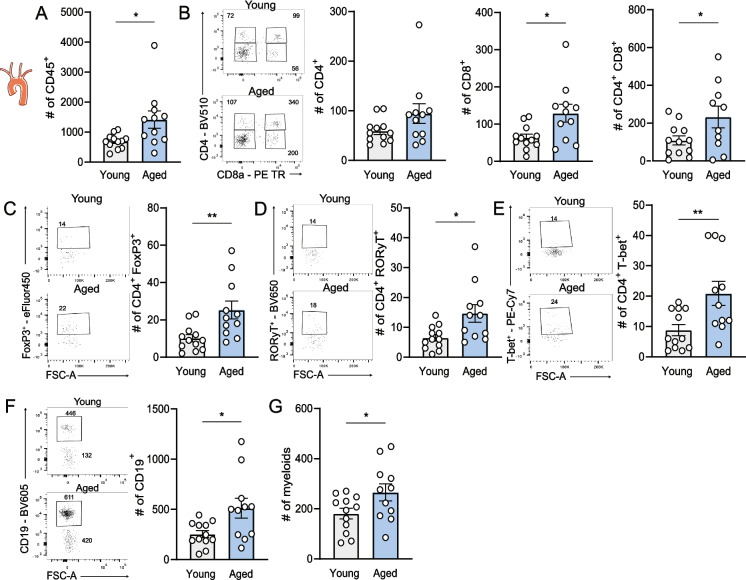
Elevated numbers of immune cells in atherosclerotic aortic arch of aged mice. At sacrifice, aortic arches of young and aged WT mice were digested and flow cytometry was used to determine the number of **(A)** total CD45^+^ immune cells, **(B)** CD4^+^, CD8^+^ and CD4^+^ CD8^+^ double positive T cells, **(C)** Th17 (RORγT^+^), **(D)** Th1 (T-bet^+^), **(E)** Tregs (FoxP3^+^), **(F)** (CD19^+^) B cells and **(G)** myeloid cells (CD19^−^ CD4^−^ CD8.^−^). Representative flow cytometry dot plots are shown. Data are from *n* = 11–13 mice per group. Mean ± SEM plotted. *P < 0.05, **P < 0.01

Concomitant with larger, more advanced plaques in aged mice, we also revealed that the number of leukocytes, defined by CD45 expression, was significantly increased in the atherosclerotic aortic arch of aged mice compared to young mice (young: 690 ± 68 cells vs. aged: 1418 ± 289 cells, *p* < 0.05) (Fig. 2A). We further explored if specific leukocyte populations were increased and observed an elevation in the number of aortic CD8^+^ T cells (young: 64 ± 9 cells vs. aged: 129 ± 24 cells, *p* < 0.05) and CD4^+^ CD8^+^ double positive T cells (young: 108 ± 24 cells vs. aged: 232 ± 57 cells, *p* < 0.05) (Fig. 2B) in aged mice. Although the number of total CD4^+^ T cells was not altered between the age groups, the number of effector T cell subsets that accumulated in the atherosclerotic arch, including regulatory T cells (FoxP3^+^) (Fig. 2C), Th1 cells (T-bet^+^) (Fig. 2D) and Th17 cells (RORγT^+^) (Fig. 2E) was significantly increased in aged mice. In addition, CD19^+^ B cells (young: 253.80 ± 35.19 cells vs. aged: 514 ± 99 cells, *p* < 0.05) (Fig. 2F) and myeloid cells (young: 181 ± 21 cells vs. aged: 266 ± 34 cells, *p* < 0.05), were increased in the aortic arch of aged mice (Fig. 2G).

### Reduced CD4+ to CD8+ T cells ratio in aged atherosclerotic mice

**Fig. 3:**
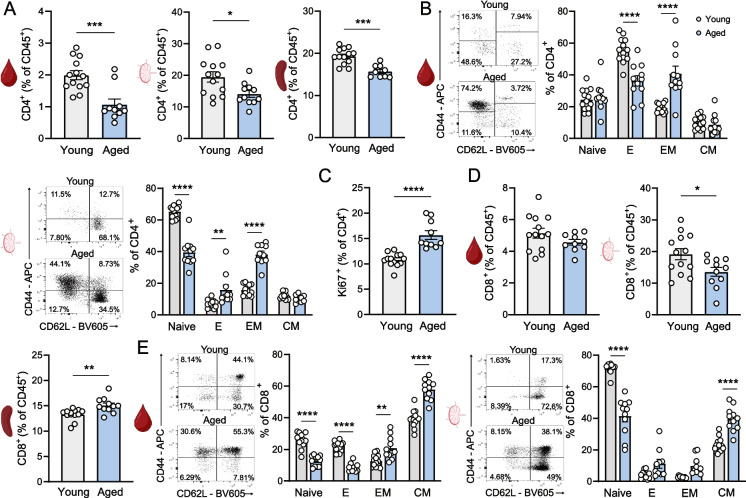
CD4^+^ and CD8^+^ T cells in aged atherosclerotic mice. At sacrifice, leukocytes from the circulation, paraaortic lymph nodes (PALN) and spleen of young and aged WT mice were analyzed with flow cytometry to assess the **(A)** frequency of CD4^+^ T cells. **(B)** Naïve (Naive: CD44^−^CD62L^+^), effector-like (E: CD44^−^CD62L^−^), central-memory (CM: CD44^+^CD62L^+^) and effector-memory (EM: CD44^+^CD62L^−^) T cells were quantified as a percentage of CD4^+^ T cells in the blood and PALN. CD4^+^ T cells were further analyzed for **(C)** the proliferation marker Ki-67 in the PALN. Flow cytometry was also used to measure the **(D)** frequency of CD8^+^ T cells in the blood, paraaortic lymph nodes and spleen. **(E)** Naïve, effector-like, central-memory and effector-memory T cells were also quantified as percentage of CD8.^+^ T cells in the blood and PALN. Data are from *n* = 11–13 mice per group. Mean ± SEM plotted. *P < 0.05, **P < 0.01, ***P < 0.001, ****P < 0.0001

In contrast to local accumulation of T cells in the atherosclerotic aorta, the frequency of CD4^+^ T cells was reduced in the circulation (young: 1.99 ± 0.14% vs. aged: 1.07 ± 0.17%, *p* < 0.001), draining paraaortic lymph nodes (young: 19.52 ± 1.65% vs. aged: 14.12 ± 1.07%, *p* < 0.05) and spleen (young: 19.32 ± 0.53% vs. aged: 15.71 ± 0.49%, *p* < 0.001) of aged mice compared to young mice (Fig. 3A), which is in line with an age-associated decline in lymphoid output. Within the CD4^+^ T cell compartment, aged atherosclerotic mice show increased effector (memory) T cells at the cost of naïve T cells (Fig. 3B, Fig. S1A). In accordance with enhanced immune cell infiltrates in the aorta, CD4^+^ T cells in the heart draining paraaortic lymph nodes of aged mice show more vigorous proliferation compared to those of young mice as identified by Ki-67 expression (young: 10.80 ± 0.37% vs. aged: 15.67 ± 0.86%, *p* < 0.0001) (Fig. 3C). No differences were found in Ki-67 expression in the blood and spleen remained unaffected (Fig. S1B). In contrast to CD4^+^ T cells, the percentage of circulating CD8^+^ T cells did not differ between the age groups (Fig. 3D). While the percentage of CD8^+^  T cells in the paraaortic lymph nodes was decreased, a slight increase was observed in the spleen of aged mice (Fig. 3D). Similar to the CD4^+^ T cell population, we observed an increase in the effector-memory and central-memory subsets paired with a twofold reduction in naïve lymphocytes in aged mice compared to young (Fig. 3E, Fig. S1C) within the CD8^+^ T cell population in these organs. No statistical differences were observed in CD8^+^ T cell proliferation (Fig. S1D).

### Extreme effector CD4^+^ T cell phenotype in aged mice

**Fig. 4:**
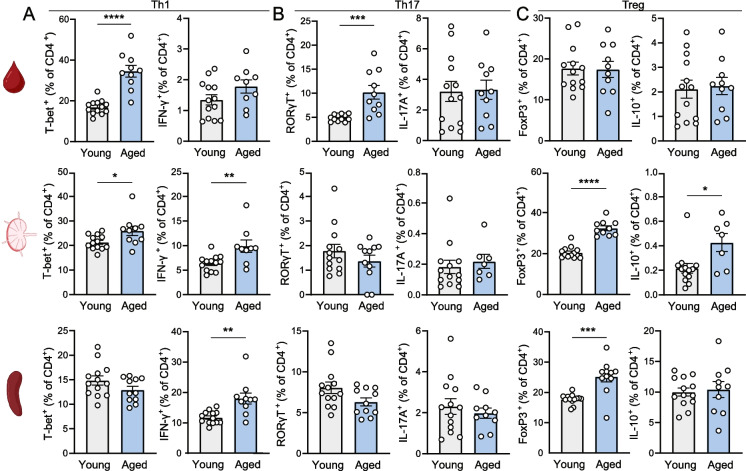
Shift towards more extreme effector CD4^+^ T cell phenotype upon aging. At sacrifice, CD4^+^ T cells from the circulation, paraaortic lymph nodes (PALN) and spleen of young and aged WT mice were analyzed with flow cytometry to determine the percentage of **(A)** Th1 cells (T-bet^+^). In addition, single cell suspensions were stimulated for 5 h with PMA, ionomycin and Brefeldin A after which intracellular IFNγ was measured. Flow cytometry was also used to measure the percentage of **(B)** Th17 cells (RORγT^+^) and intracellular IL-17A, and **(C)** regulatory T cells (FoxP3.^+^) and intracellular IL-10. Data are from *n* = 11–13 mice per group. Mean ± SEM plotted. *P < 0.05, **P < 0.01, ***P < 0.001, ****P < 0.0001

Next, we provided a more detailed analysis of the effector CD4^+^ T cell phenotype, as different T helper subsets have distinct roles in atherosclerosis progression, dependent on their hallmark cytokine secretion. In line with elevated Th1 cells in the atherosclerotic aortic arch of aged mice, we observed an increase in the frequency of pro-atherogenic T-bet^+^ Th1 cells in the circulation and paraaortic lymph nodes of aged compared to young mice (blood young: 16.86 ± 0.95% vs. aged: 34.50 ± 2.89%, *p* < 0.0001, PALN young: 21.32 ± 0.80% vs. aged: 25.93 ± 1.91%, *p* < 0.05) (Fig. 4A) Similarly, T cell stimulation with PMA and ionomycin resulted in a significantly elevated frequency of IFNγ-expressing CD4^+^ T cells, the hallmark Th1 cytokine, in the draining paraaortic lymph nodes (young: 6.40 ± 0.44% vs. aged: 9.90 ± 1.25%, *p* < 0.01) and spleen (young: 11.73 ± 0.57% vs. aged: 17.99 ± 5.74%, *p* < 0.01) of aged mice (Fig. 4A). In addition, the median fluorescent intensity (MFI) of IFNγ on CD4^+^ T cells was elevated in aged compared to young atherosclerotic mice (Fig. S2A). Th17 differentiation was only upregulated in the circulation of aged mice (young: 4.91 ± 0.20% vs. aged: 10.19 ± 1.42%, *p* < 0.001), but not in the draining paraaortic lymph nodes and spleen (Fig. 4B). In line with this observation, the frequency and expression levels of IL-17A per cell were not significantly affected upon aging (Fig. S2B). Furthermore, we evaluated the frequency of anti-atherogenic regulatory T cells (Tregs) in young and aged atherosclerotic mice (Fig. 4C). Aging has previously been associated with an accumulation in Tregs and increased FoxP3 expression as a possible response to sustained chronic inflammation [35, 36]. In accordance, we observed an increase in FoxP3^+^ Tregs in aged draining paraaortic lymph nodes and spleens (PALN young: 20.48 ± 0.78% vs. aged: 32.42 ± 1.17%, *p* < 0.0001, spleen young: 17.76 ± 0.46% vs. aged: 25.21 ± 1.70%, *p* < 0.001), with a concomitant increase in IL-10-producing CD4^+^ T cells in the draining paraaortic lymph nodes (young: 0.22 ± 0.04% vs. aged: 0.43 ± 0.07%, *p* < 0.05). Together with the increased expression of IL-10 per CD4^+^ T cell in the draining lymph nodes and spleen (Fig. S1C), this suggests an anti-inflammatory counterbalance to the increase in pro-inflammatory Th1-associated IFN-γ in aged mice.

### Increased cellular senescence in aged mice

**Fig. 5:**
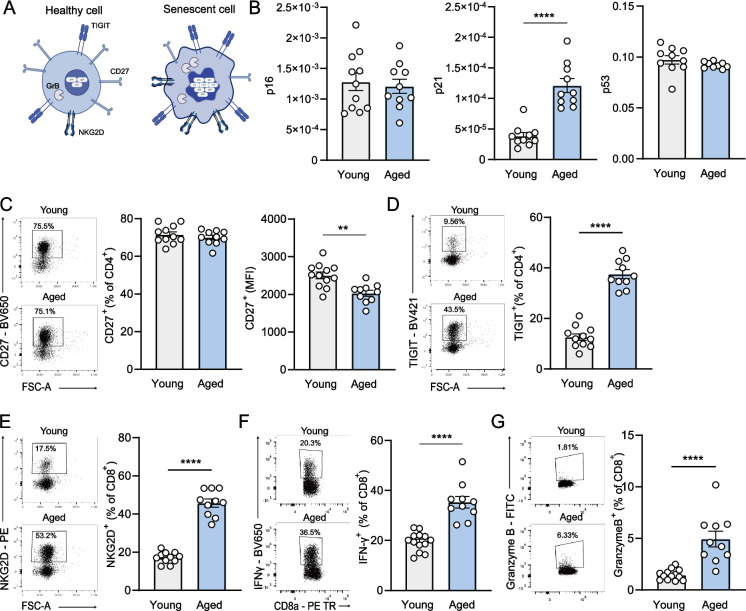
Senescent T cell phenotype in the spleens of aged mice. Splenocytes were isolated from young and aged WT mice and investigated for **(A)** senescent cell features. The **(B)** mRNA expression of p16, p21 and p53 was assessed using qPCR. The expression and mean fluorescence intensity (MFI) of **(C)** CD27 on CD4^+^ T cells, and the expression of **(D)** TIGIT on CD4^+^ T cells and **(E)** NKG2D on CD8^+^ T cells were measured using flow cytometry. CD8.^+^ T cells were stimulated with PMA and ionomycin for 5 h after which **(F)** IFN-γ and **(G)** Granzyme B (GzmB) expression were measured using flow cytometry. Data are from *n* = 10–13 mice per group. Mean ± SEM plotted. **P < 0.01, ***P < 0.001, ****P < 0.0001. Created in BioRender. De Mol, J. (2025) https://BioRender.com/d4bbq44

In addition to a more extreme T cell phenotype in aged mice, aging is associated with accumulation of senescent cells [37]. Senescent cells secrete various pro-inflammatory factors, including cytokines, chemokines, proteases and growth factors, which may contribute to a pro-atherogenic microenvironment [38]. We examined the expression of kinase inhibitors p16, p21 and p53 in the spleen, which promote the senescence-related cell-cycle arrest (Fig. 5A), and found almost a threefold increase in p21 expression in aged mice (young: 3.86 ± 0.56 × 10^–5^ reads per million (RPM) vs. aged: 12.10 ± 1.14 × 10^–5^ RPM, *p* < 0.0001) (Fig. 5B). Loss of CD27 expression on T lymphocytes is associated with immunosenescence [39] and we conformingly revealed a decrease in the expression of, but not frequency of, CD27 (young: 2504 ± 90.27 MFI vs. aged: 2033 ± 79.32 MFI, *p* < 0.01) on aged CD4^+^ T cells (Fig. 5C). Furthermore, the frequency of splenic CD4^+^ T cells that expressed the coinhibitory receptor TIGIT was highly increased in aged mice (young: 12.61 ± 1.29% vs. aged: 37.55 ± 1.77%, *p* < 0.0001) (Fig. 5D). Although binding of TIGIT with its ligand generally induces T cell suppression, TIGIT^+^ T cells accumulate upon aging and its increased expression is related to T cell exhaustion and senescence [40, 41]. The frequency of NKG2D, a receptor that was found to bind to senescent cells [42, 43], was 2.6-fold higher expressed on aged compared to young CD8^+^ T cells (young: 17.19 ± 0.89% vs. aged: 45.78 ± 2.1%, *p* < 0.0001) (Fig. 5E) indicating a senescent-related phenotype of T lymphocytes in the aged mice. Upon stimulation, these aged splenic CD8^+^ T cells showed increased expression of the pro-inflammatory cytokine IFNγ (young: 19.55 ± 1.04% vs. aged: 35.36 ± 2.34%, *p* < 0.0001) (Fig. 5F) and cytotoxic factor granzyme B (young: 1.47 ± 0.14% vs. aged: 4.94 ± 0.76%, *p* < 0.0001) (Fig. 5G), providing further evidence that T cell senescence contributed to accelerated atherosclerosis development observed in aged mice.

### Altered humoral immune responses upon aging

**Fig. 6:**
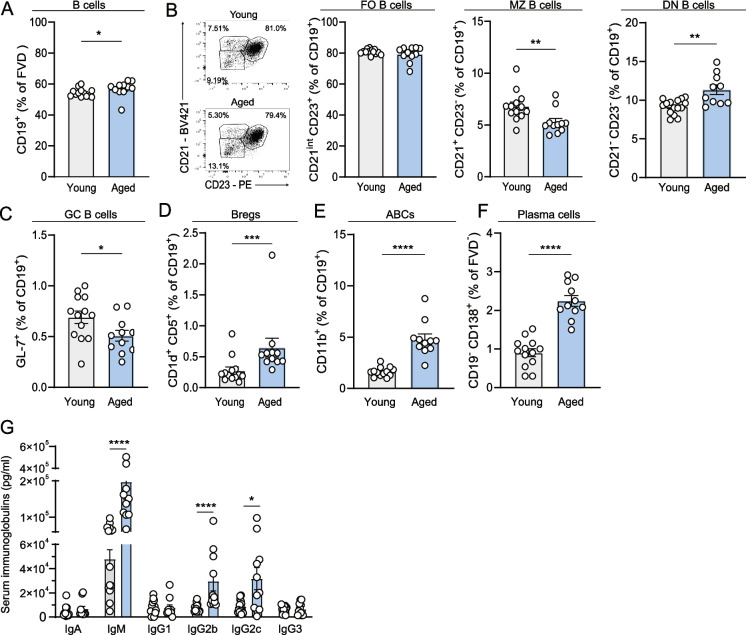
Age-associated alterations in splenic B cell compartment. At sacrifice, splenocytes of young and aged WT mice were analyzed with flow cytometry to determine the percentage of **(A)** B cells (CD19^+^) cells. **(B)** Follicular (CD21^int^ CD23^+^), marginal zone (CD21^+^ CD23^−^), double negative (CD21^−^ CD23^−^), **(C)** germinal center (GL-7^+^), **(D)** regulatory (CD1d^+^ CD5^+^) and **(E)** age-associated (CD11b^+^) B cells were quantified as percentage of B cells. Flow cytometry was also used to determine the percentage of splenic **(F)** plasma cells (CD19^−^ CD138.^+^). **(G)** A legendplex assay was performed to measure immunoglobulins in the serum of mice. Data are from *n* = 11–13 mice per group. Mean ± SEM plotted. *P < 0.05, **P < 0.01, ***P < 0.001, ****P < 0.0001

We reported elevated numbers of B cells in the aortic arch of aged AAV-PCSK9 mice, which can contribute to atherosclerosis development via the production of antibodies, cytokines and cross-talk with T cells [44]. We therefore further investigated the B cell landscape in the spleen, where B cells become activated and can differentiate into antibody-secreting plasma cells. We first assessed the frequency of total splenic B cells and observed a slight increase in aged compared to young mice (young: 54.38 ± 0.75% vs. aged: 56.94 ± 1.55%, *p* < 0.05) (Fig. 6A). While we did not observe a difference in the percentage of follicular B cells (CD21^int^ CD23^+^) (Fig. 6B), spleens of aged mice contained relatively less marginal zone B cells (CD21^+^ CD23^−^ in young: 6.82 ± 0.40% vs. aged: 5.28 ± 0.35%, *p* < 0.01) and more CD21^−^ CD23^−^ B cells (young: 2.91 ± 0.54% vs. aged:4.96 ± 0.59%, *p* < 0.01) compared to young spleens. Within the double negative B cell population, the percentage of germinal center B cells (GL-7^+^) decreased (young: 0.69 ± 0.06% vs. aged: 0.51 ± 0.05, *p* < 0.05) (Fig. 6C), while the frequency of CD1d^+^ CD5 regulatory (young: 0.28 ± 0.06% vs. aged: 0.65 ± 0.15%, *p* < 0.001) (Fig. 6D) and age-associated B cells (ABCs) (young: 1.63 ± 0.13% vs. aged: 4.80 ± 0.51%, *p* < 0.0001) (Fig. 6E) was increased in aged compared to young mice. Since age-associated B cells have a pro-inflammatory signature [32] and can give rise to long-lived plasma cells [45–47], we investigated the percentage of CD138^+^ plasma cells and found more than a twofold increase in aged spleens (young: 0.90 ± 0.10% vs. aged: 2.25 ± 0.14%) (Fig. 6F). In line with these findings, we measured higher levels of IgM (young: 0.46 ± 0.08 × 10^5^ pg/mL vs. aged: 1.97 ± 0.47 × 10^5^ pg/mL), IgG2b (young: 0.71 ± 0.097 × 10^4^ pg/mL vs. aged: 2.95 ± 0.78 × 10^4^ pg/mL) and IgG2c (young: 0.80 ± 0.01 × 10^4^ pg/mL vs. aged: 3.17 ± 0.09 × 10^4^ pg/mL), but not IgA, IgG1 and IgG3, in the serum of aged AAV-PCSK9 mice (Fig. 6G).

## Discussion

Aging is one of the most dominant risk factors of ASCVD and is associated with a gradual functional decline of the immune system. Yet, the majority of preclinical atherosclerosis studies are performed in young mice and do not take immunosenescence into account. To assess the effects of immunosenescence on atherosclerosis development, we employed administration of a rAAV-PCSK9 and WTD feeding to induce atherosclerosis development in young versus aged mice and showed that age-associated pro-inflammatory immune alterations contribute to aggravated atherosclerosis upon aging.

In line with our observed acceleration in atherosclerosis development in aged rAAV-PCSK9-treated C57Bl/6 mice, Tyrell et al*.* showed increased atherosclerosis development in the brachiocephalic artery of aged C57Bl/6 mice transfected with rAAV-PCSK9 compared to young mice [48]. Although relatively initial, plaques present in aged mice showed a more advanced phenotype compared to those of young mice, as indicated by the increase in plaque collagen content. This increase in collagen may be attributed to aged vascular smooth muscle cells, which exhibit excessive collagen deposition [49]. Moreover, the demonstrated elevation in immune cell infiltrates might be the consequence of a disrupted barrier function due to increased endothelial cell senescence in aged arteries of humans and wildtype mice [50, 51]. Additionally, macrophages from C57BL/6 mice showed increased chemokine release [52], and age-associated adaptive immune cells displayed elevated chemokine receptor expression in *Ldlr*^*−/−*^ mice [53], potentially contributing to increased T- and B cell infiltration in the aged aorta. Furthermore, the pro-inflammatory mediators secreted by aged immune cells can further ensue endothelial dysfunction and immune cell infiltration and promote pro-atherogenic immune cell responses [54], which is in line with the observed elevation of T-bet^+^ and RORγT^+^ CD4^+^ T cells in the aorta. Similar to atherosclerotic aortas in *Ldlr*^*−/−*^ mice [32], we detected a robust increase in the number of CD4^+^ CD8^+^ double positive T cells. Although the role of these T cells in CVD is not intensively studied, high levels of CD4^+^ CD8^+^ double positive T cells have been identified in autoimmune and chronic inflammatory disorders [55, 56].

Systemically, we detected a robust decrease in the frequency of CD4^+^ T cells in aged mice, likely attributed to thymic involution and a reduced lymphoid output from the bone marrow [57, 58]. Not surprisingly, within the CD4^+^ and CD8^+^ T cell compartment from aged atherosclerotic mice, we found hallmarks of T cell aging and a local and systemic increase in the frequency of Th1 cells, which can contribute to the accelerated atherosclerosis progression in aged mice [59, 60]. This elevation was accompanied with an upregulated mean pro-atherogenic IFNγ expression in both CD4^+^ and CD8^+^ T cells, which can be the result of, and further stimulate, overactivated cell signaling pathways in immunosenescent T cells [61, 62]. Increased granzyme B expression in CD8^+^ T cells has also been described to correlate with aging and senescence in humans [63, 64], and this cytolytic factor is increased in CVD patients [65, 66]. In addition, we measured higher circulating Th17 cell percentages, which have also been associated with aggravated atherogenesis [67].

In the B cell compartment, expansion of CD23^−^ CD21^−^ B cells, of which ABCs comprise the largest subset, can contribute to the observed accelerated atherosclerosis in aged mice. ABCs accumulate upon aging and in autoimmune diseases and contribute to inflammation via pro-inflammatory cytokine and antibody secretion [20]. Recently, we and others detected ABCs in plaques of aged atherosclerotic mice, which showed a pro-atherogenic signature and highly express plasma cell differentiation genes [32, 68, 69]. Accordingly, we observed elevated plasma cells and circulating IgM, IgG2b and IgG2c in aged rAAV-PCSK9 mice. Although IgM is described to dampen inflammation and atherosclerosis progression [70], IgGs, can stimulate atherosclerosis development and have been associated with enhanced plaque stability [71], which can attribute to the more advanced lesion phenotype in aged mice.

In summary, our research provides a more in-depth understanding of immunosenescence in atherosclerosis. We demonstrate that aging is associated with an exacerbated pro-inflammatory immune landscape, contributing to aggravated atherosclerosis development upon acute hypercholesterolemia. Future studies must provide additional mechanistic insights into the drivers and effects of modulating immunosenescence in atherosclerosis. Targeting of age-related immune dysregulation could represent a promising novel anti-atherosclerosis therapy.

## Supplementary Information

Below is the link to the electronic supplementary material.

Supplementary file1 (PDF 1.66 KB)
