# Supplementary material for: Immunosenescence accelerates atherosclerosis development in AAV-PCSK9 mouse model
Source: GeroScience. 2025 Jul 3;47(5):6549–63. doi: 10.1007/s11357-025-01768-6 (PMC12634928; doi:10.1007/s11357-025-01768-6)
Supplement: Supplementary file 1 — Supplementary file1 (PDF 1.66 KB) [file 11357_2025_1768_MOESM1_ESM.pdf]

Supplemental material

Immunosenescence accelerates atherosclerosis development in AAV-PCSK9 mouse model

Running title: Immunosenescence accelerates atherosclerosis development

Jill de Mol, Msc.[1], Virginia Smit, PhD.[1], Mireia N.A. Bernabé Kleijn, BSc.[1], Peter J. van Santbrink[1], Ilze Bot, PhD[1], Amanda C. Foks, PhD[1]

[1] Leiden Academic Centre for Drug Research, Division of BioTherapeutics, Leiden University, Einsteinweg 55, 2333 CC Leiden, The Netherlands.

Correspondence to:

Amanda C. Foks, PhD

Division of BioTherapeutics

Leiden University, LACDR

Einsteinweg 55, 2333CC Leiden

The Netherlands

Tel; +31 (0) 71-5276213

a.c.foks@lacdr.leidenuniv.nl

Supplemental Table 1. Antibodies for flow cytometry

Marker	Fluorochrome	Clone	Cat. No.	Working concentration	Source
CD45	AF700	30-F11	103128	1:1000	Biolegend
CD4	BV510	RM4-5	100559	1:1000	Biolegend
CD19	BV605	6D5	115540	1:500	Biolegend
CD8a	PE-Texas red	53-6.7	MCD0817	1:1500	Invitrogen
CD44	APC	IM7	17-0441-82	1:1000	eBioscience
CD62L	BV605	MEL-14	104438	1:500	Biolegend
FoxP3	eFluor 450	FJK-16s	48-5773-82	1:500	eBioscience
RORγT	BV650	Q31-378	564722	1:300	BD Horizon
T-bet	PE-Cy7	eBio4B10	25-5825-82	1:1000	eBioscience
Ki-67	FITC	SolA15	11-5698-82	1:1000	eBioscience
IL-17A	PE	TC11-18H10.1	506904	1:100	Biolegend
IFNγ	BV650	XMG1.2	505832	1:500	Biolegend
IL-10	APC	JES5-16E3	505010	1:100	Biolegend
Granzyme B	FITC	QA16A02	372206	1:400	Biolegend
CD21	BV421	7E9	123422	1:400	Biolegend
CD23	PE	B3B4	101607	1:500	Biolegend
CD5	PerCP	53-7.3	100616	1:400	Biolegend
CD1d	FITC	1B1	123507	1:400	Biolegend
GL7	PE-Cy7	GL-7	144620	1:500	Biolegend
CD11b	PE	M1/70	101208	1:500	Biolegend
CD11c	FITC	N418	117306	1:500	Biolegend
CD138	BV650	281-2	142508	1:400	Biolegend
CD8a	AF700	53-6.7	100730	1:500	Biolegend
NKG2D	PE	CX5	130207	1:500	Biolegend
TIGIT	BV421	1G9	142111	1:400	Biolegend
CD27	BV650	LG-3A10	124233	1:500	Biolegend
CD16/32 (Fc Block)	-	93	553142	1:250	BD Biosciences
Fixable viability dye	eFluor-780	-	65-0865-18	1:2000	eBioscience

Supplemental Table 2. Primers for qPCR

Gene	Forward primer	Reverse primer
p16	tctttgtgtaccgctgggaacgtc	agctaagaagaaaaaggcgggctgag
p21	ctgtcttgcactctggtgtctgagc	aagaccaatctgcgcttggagtgat
p53	taaacgcttcgagatgttccgggag	aggccccactttcttgaccattgtt
36B4	taaccctgaagtgctcgacatcacag	gacgcgcttgtacccattgatgatg
Ppia	gaaaactttcgagctctgagcactgg	ctgccgccagtgccattatgg
Actin-beta	cttctttgcagctccttcgttgccg	aatacagcccggggagcatcgtc

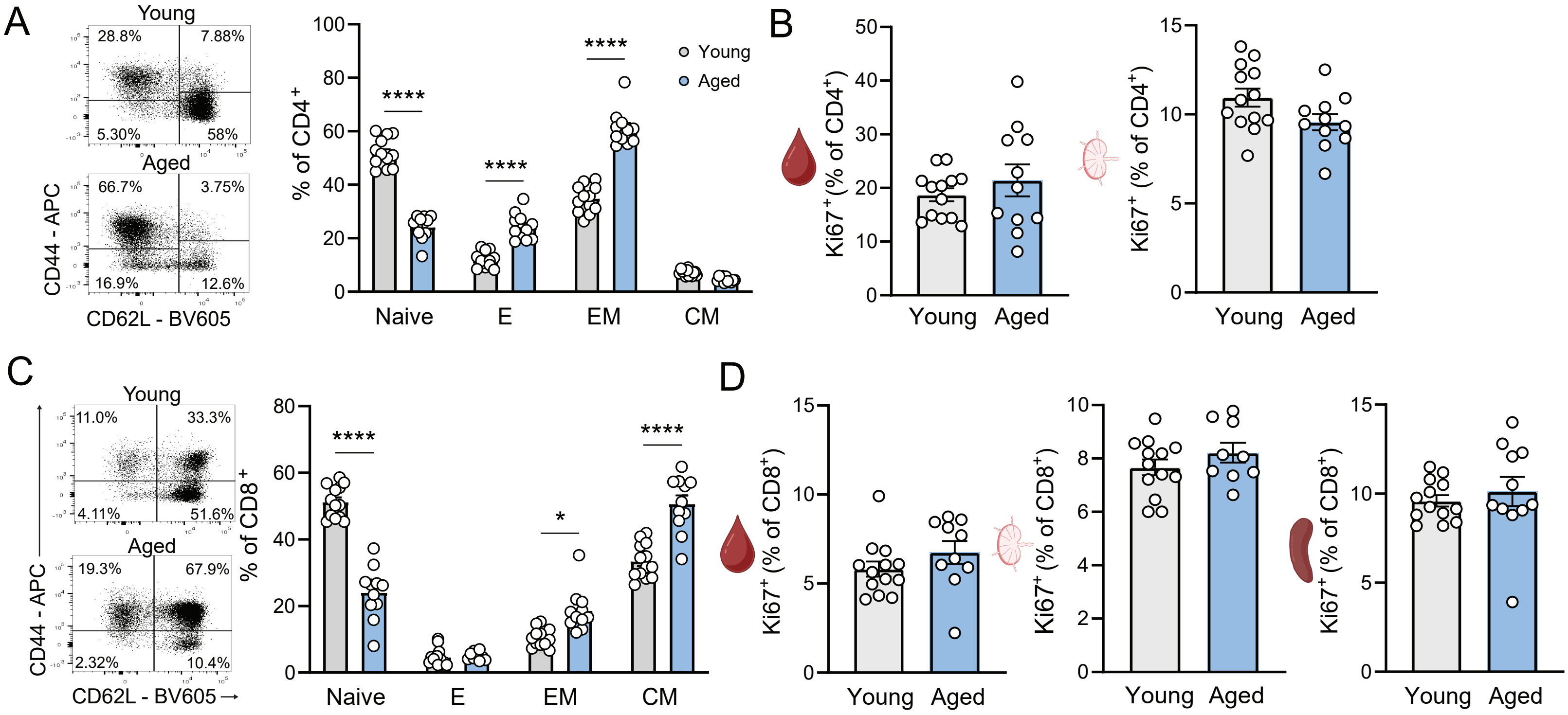

Supplemental Figure 1. CD4$^+$ and CD8$^+$ T cells in aged atherosclerotic mice. At sacrifice, leukocytes from the circulation, paraaortic lymph nodes (PALN) and spleen of young and aged WT mice were analyzed with flow cytometry to assess the **(A)** Naïve (Naive: CD44$^-$CD62L$^+$), effector-like (E: CD44$^-$CD62L$^-$), central-memory (CM: CD44$^+$CD62L$^+$) and effector-memory (EM: CD44$^+$CD62L$^-$) T cells were quantified as a percentage of CD4$^+$ T cells in the spleen. CD4$^+$ T cells were further analyzed for **(B)** the proliferation marker Ki-67 in the blood and PALN. Flow cytometry was also used to measure the **(C)** Naïve, effector-like, central-memory and effector-memory T cells were also quantified as percentage of CD8$^+$ T cells in the spleen. CD8$^+$ T cells were further analyzed for **(D)** Ki-67. Data are from n=11-13 mice per group. Mean ± SEM plotted. *P<0.05, ****P<0.0001.

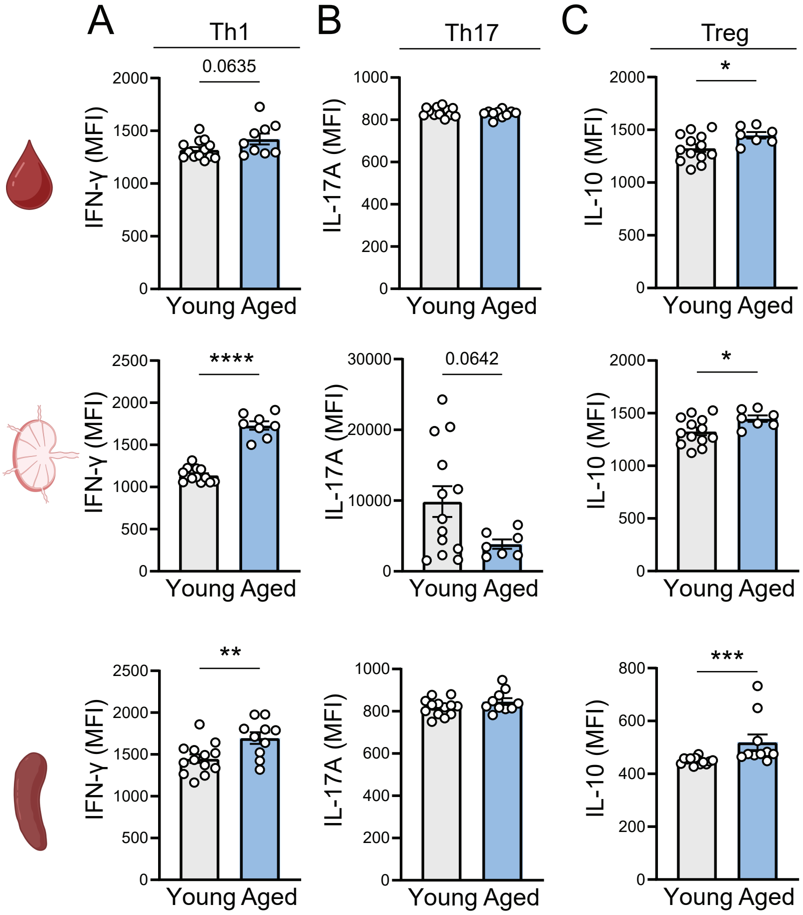

Supplemental Figure 2. Elevated cytokine expression on CD4⁺ T cells of aged mice. $CD4^+$ T cells from the circulation, paraaortic lymph nodes (PALN) and spleen of young and aged WT mice were analyzed with flow cytometry for the mean expression of **(A)** IFNγ, **(B)** IL-17A and **(C)** IL-10. Data are from n=11-13 mice per group. Mean ± SEM plotted. *P<0.05, **P<0.01, ***P<0.001, ****P<0.0001.
